# Chromosome-level genome assembly of the predatory stink bug *Arma custos*

**DOI:** 10.1038/s41597-024-03270-8

**Published:** 2024-04-23

**Authors:** Yuqin Wang, Yunfei Luo, Yunkang Ge, Sha Liu, Wenkai Liang, Chaoyan Wu, Shujun Wei, Jiaying Zhu

**Affiliations:** 1 Key Laboratory of Forest Disaster Warning and Control of Yunnan Province, Southwest Forestry University, Kunming, 650224 China; 2 Institute of Plant Protection, Beijing Academy of Agriculture and Forestry Sciences, Beijing, 100091 China; 3 Key Laboratory for Forest Resources Conservation and Utilization in the Southwest Mountains of China, Ministry of Education, Southwest Forestry University, Kunming, 650224 China

**Keywords:** Entomology, Genome

## Abstract

The stink bug *Arma custos* (Hemiptera: Pentatomidae) is a predatory enemy successfully used for biocontrol of lepidopteran and coleopteran pests in notorious invasive species. In this study, a high-quality chromosome-scale genome assembly of *A. custos* was achieved through a combination of Illumina sequencing, PacBio HiFi sequencing, and Hi-C scaffolding techniques. The final assembled genome was 969.02 Mb in size, with 935.94 Mb anchored to seven chromosomes, and a scaffold N50 length of 135.75 Mb. This genome comprised 52.78% repetitive elements. The detected complete BUSCO score was 99.34%, indicating its completeness. A total of 13,708 protein-coding genes were predicted in the genome, and 13219 of them were annotated. This genome provides an invaluable resource for further research on various aspects of predatory bugs, such as biology, genetics, and functional genomics.

## Background & Summary

The stink bug *Arma custos* (Fabricius, 1794) (Hemiptera: Pentatomidae) is synonymous with *Arma chinensis* (Fallou, 1881), which has been recorded in China, Mongolia and Korea, as well as central and southern Europe (except the British Islands) and the neighboring parts of the Middle East^1,2^. Both nymphs and adults of this zoophytophagous bug can predate many agricultural and forestry pests belonging to the orders of Coleoptera, Lepidoptera, Hemiptera and Hymenoptera by utilizing a venomous cocktail produced by the salivary gland to capture and digest preys^3,4^. It can be easily mass-reared using artificial diet in a factory and exhibits strong adaptability to diverse ecological niches, enabling its successful use as a commercialized biocontrol agent^3,5^. Notably, it has shown effective management of notorious invasive pests such as the fall webworm *Hyphantria cunea*, the Colorado potato beetle *Leptinotarsa decemlineata*, and the fall armyworm *Spodoptera frugiperda* through the augmentative release^6–8^. However, limited attention has been given to the investigation of the biological characteristics^9–11^, artificial rearing methods^3,5,12^, chemoecology^13^, response to temperature and drought stresses^8,14–17^, and developmental regulation by miRNA^18^ of this predatory bug. In terms of its genetic information, only the mitochondrial genome and several transcriptomic datasets are available as the current genetic resources^13,15,16,18,19^. Obtaining high-quality genome for providing a whole set of gene resources of *A. custos* will greatly facilitate a wide range of biological researches and allow further investigations, such as population genetic diversity, venomics, adaptive evolution, and comparative genomics.

In this study, we have assembled a chromosome-level genome of *A. custos* by combining PacBio HiFi sequencing and High-throughput chromosome conformation capture (Hi-C) technologies. The genome assembly allowed us to identify repeat sequences and protein-coding genes. Predicted genes were annotated. The generated genomic resources will facilitate to the investigation of this predatory bug.

## Methods

### Sample collection and rearing

The population of *A. custos* used in this study originated from a colony collected in the suburb of Kunming, Yunnan Province, China. These bugs have been maintained in our laboratory for more than 20 generations. They were fed with larvae of the yellow mealworm *Tenebrio molitor*, the greater wax moth *Galleria mellonella*, and the fall armyworm *S. frugiperda*. Cages measuring 40 cm × 40 cm × 40 cm, constructed with Nylon netting (44 × 32 mesh) on all sides, were used to rear the bugs. Each cage housed approximately 100 bugs. Soybean plants were also provided in the cage for feeding and perching. The bugs were reared at a constant temperature of 25 ± 1 °C, 70 ± 5% relative humidity, and a photoperiod of 14 L:10D.

### Sequencing

Genomic DNA was extracted from one newly emerged male adult using the QIAamp DNA Mini Kit (Qiagen, Hilden, Germany). Total RNA was isolated from various adult tissues including different glands of the salivary venom apparatus (anterior main gland, posterior main gland, and accessary gland), gut and residual body (adult deprived of salivary venom apparatus and gut). The integrity and contamination of the DNA and RNA were assessed on a 1% agarose gel. The purity of the DNA and RNA was measured with a NanoDrop 2000c spectrophotometer (Thermo Fisher Scientific, Waltham, MA, USA). The DNA and RNA concentration of was determined using the Qubit DNA Assay Kit in Qubit 3.0 Flurometer (Invitrogen, Carlsbad, CA, USA).

For short-read genomic and transcriptome sequencing, the library with an insert size of 350 bp was constructed using the NEBNext Ultra DNA Library Prep Kit (Illumina, San Diego, CA, USA) following manufacturer’s recommendations. This library was then sequenced on the Illumina NovaSeq 6000 platform (Illumina, San Diego, CA, USA). The genomic short-read data yielded from the Illumina NovaSeq 6000 platform amounted to 74.86 Gb with a Q20 value of 96.56% and a Q30 value of 90.84% (Table 1). A total of 72.86 Gb transcriptomic data were generated, which have Q20 values over 96.56% and Q30 values more than 90.84%.

**Table 1:** Statistics of sequencing data for genome assembly and annotation.

Library type	Sequencing platform	Sample	Reads number	Raw data (Gb)	NCBI SRA accession no.
Genome	Illumina NovaSeq 6000	Male adult	249,518,674	74.86	SRR25498178
Genome	PacBio sequel II	Male adult	2,299,735	34.41	SRR25503034
Hi-C	Illumina NovaSeq 6000	Male adult	8,661,026	163.62	SRR25518321
Transcriptome	Illumina NovaSeq 6000	Anterior main gland of male	42,024,044	12.61	SRR25541878 SRR25541877
Transcriptome	Illumina NovaSeq 6000	Posterior main gland of male	45,376,382	13.61	SRR25541873 SRR25541872
Transcriptome	Illumina NovaSeq 6000	Accessary gland of male	42,227,792	12.67	SRR25541880 SRR25541879
Transcriptome	Illumina NovaSeq 6000	Duct of accessary gland of male	43,771,219	13.13	SRR25541882 SRR25541881
Transcriptome	Illumina NovaSeq 6000	Gut of male	40,425,550	12.13	SRR25541876 SRR25541875
Transcriptome	Illumina NovaSeq 6000	Residual body of male	44,540,809	13.36	SRR25541871 SRR25541870
Transcriptome	Illumina NovaSeq 6000	Anterior main gland of female	44,301,583	13.29	SRR25541868 SRR25541867
Transcriptome	Illumina NovaSeq 6000	Posterior main gland of female	61,418,417	18.52	SRR25541864 SRR25541863
Transcriptome	Illumina NovaSeq 6000	Accessary gland of female	43,782,125	13.13	SRR25541874 SRR25541869
Transcriptome	Illumina NovaSeq 6000	Duct of accessary gland of female	44,006,115	13.2	SRR25541886 SRR25541885
Transcriptome	Illumina NovaSeq 6000	Gut of female	41,769,353	12.53	SRR25541866 SRR25541865
Transcriptome	Illumina NovaSeq 6000	Residual body of female	45,693,537	13.71	SRR25541884 SRR25541883

For PacBio HiFi long-read sequencing, the SMRTbell library was prepared with the SMRTbell Express template preparation kit 2.0 (Pacific Biosciences, Menlo Park, CA) and subsequently sequenced using the Sequel II Sequencing Kit 2.0 with SMRT Cell 8 M Tray on a PacBio sequel II instrument (Pacific Biosciences, Menlo Park, CA). In total, 34.41 Gb high-quality HiFi reads (34.85 × coverage) were obtained with an average length of 14.96 kb and an N50 length of 15.18 kb (Table 1).

The Hi-C library was generated using the restriction endonuclease Mbol following the standard protocol described previously^20^, which was sequenced on the Illumina NovaSeq 6000 platform (Illumina, San Diego, CA, USA) using a 150-bp paired-end strategy. A total of 163.62 Gb (165.72 × coverage) of raw data was generated.

### Genome survey

**Fig. 1:**
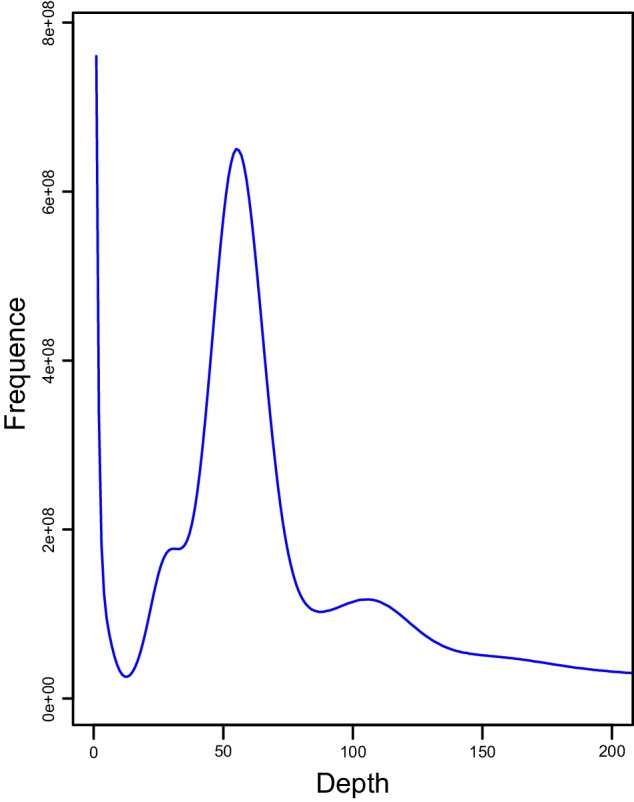
The 17-mer analysis of the genome of *Arma custos*. The X-axis represents the k-mer depth. The Y-axis indicates the k-mer frequency for a given depth.

To ensure data quality, adapter sequences and low-quality reads were removed with fastp v0.21.0^21^. The resulting clean reads were used to generate a histogram of the 17-mer distribution with Jellyfish v2.2.7 with parameters ‘count -g generators -G 4 -s 5 G -m 17 -C -t 10’^22^ (Fig. 1), followed by calculation of genome heterozygosity. Based on these analyses, the estimated genome size was determined to be 987.35 Mb, with a heterozygosity of 0.80%.

### Genome assembly

The PacBio HiFi reads were utilized to assemble the genome into contigs using hifiasm v0.16.1^23^. The assembled draft genome was polished by employing the genomic short-reads generated by Illumina NovaSeq 6000 sequencer with the NextPolish v1.4.0^24^. To identify and remove potential contaminant sequences, Kraken2 was employed against a custom database^25^. A total of 137 contigs were identified as bacteria and subsequently eliminated. The resulting draft genome was 969.02 Mb with a contig N50 of 2.11 Mb, and the GC content of 33.18% (Table 2).

**Table 2:** Statistics of the *Arma custos* genome assembly.

Features	Statistics
Total contig length (bp)	969,016,255
Number of contigs	1,142
Contig N50 size (bp)	2,105,537
Maximum contig size (bp)	11,334,306
Number of chromosomes	7
Total length of chromosomes (bp)	935,936,572
GC content (%)	33.18

### Hi-C scaffolding

**Fig. 2:**
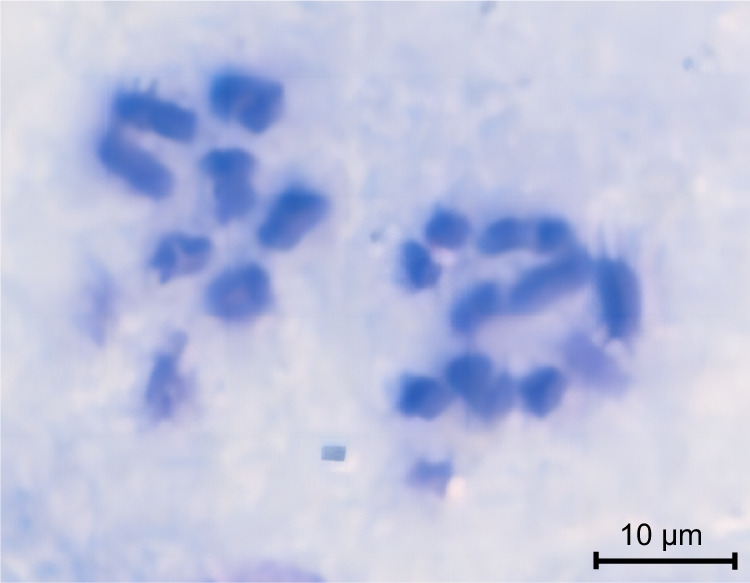
Karyotype analysis of *Arma custos* reveals a chromosome count of seven. The chromosomes from two nuclei are shown.

**Fig. 3:**
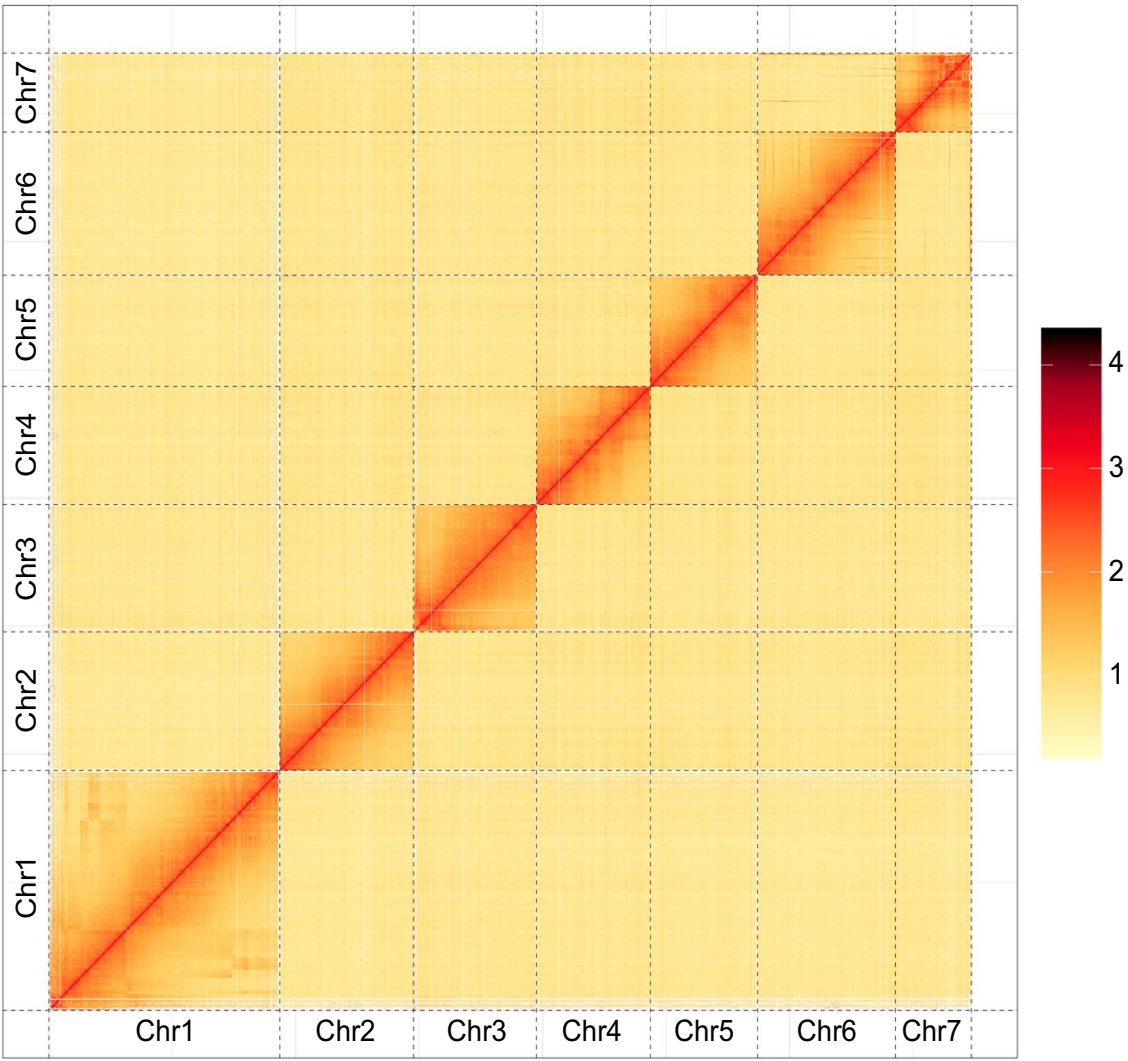
Heatmap of the Hi-C assembly of *Arma custos*. The interaction intensity of Hi-C links represents by colors shown in the left bar, ranging from yellow (low) to red (high).

**Fig. 4:**
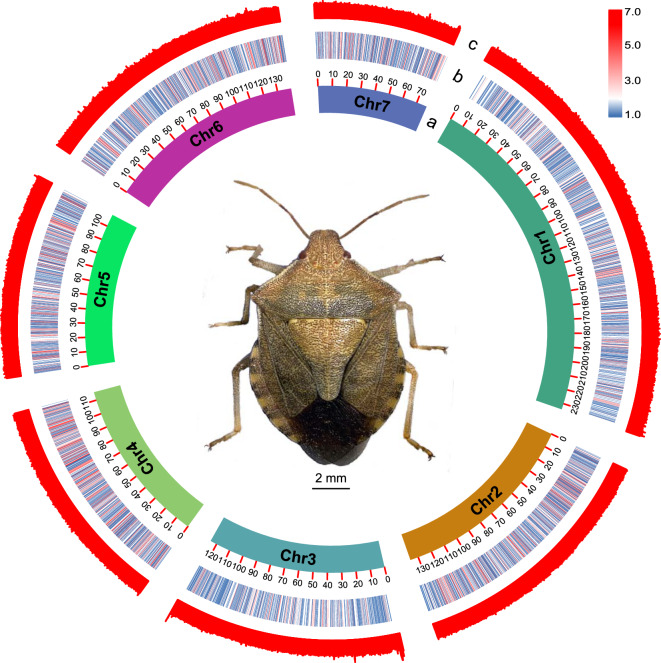
Overview of the genome characteristics of *Arma custos* in a circos plot. (**a**), length of chromosomes at the Mb scale; (**b**), gene density per Mb; (**c**), CG content per Mb.

The raw HiC data were processed using Hi-C-Pro v2.8.0^26^, followed by quality control with fastp v0.21.0^21^. The resulting data were aligned to the draft genome assembly utilizing bowtie 2 v2.2.3^27^ to obtain the uniquely mapped paired-end reads. Among the 8,661,026 reads, 4,330,513 reads were paired, with a total paired ratio of 38.70%. And a total of 1,470,719 reads were uniquely mapped to the genome, with an effect rate of 33.96%, representing valid interaction pairs. These valid interaction pairs were used to anchor the assembled contigs to near-chromosomal level using the Allhic v0.9.8^28^. Then, juicebox v1.11.08^29^ was employed for manual correction based on chromosome interaction strength, ultimately resulting a chromosome-level genome. After curation, a total of 935.94 Mb of contigs, accounting for 96.58% of the assembled draft genome, were anchored into seven chromosomes, ranging from 77.33 Mb to 234.11 Mb (Table 3). The number of anchored chromosomes matched the result of chromosome karyotype analysis following the previously reported method^30^ (Fig. 2). The final genome exhibited an N50 of 135.75 Mb. A genome-wide chromatin interaction HiC heatmap was constructed using the ggplot2 software in the R package. According to the heatmap, all chromosomes were clearly distinguishable from each other (Fig. 3). The Advanced Circos tool implanted in TBtools v1.098765^31^ was used to visualize the landscape of the chromosomes (Fig. 4).

**Table 3:** Summary of the assembled seven chromosomes of *Arma custos*.

Chr ID	Contig number	Chr length (bp)
Chr1	174	234,112,533
Chr2	138	135,751,263
Chr3	147	124,603,657
Chr4	97	115,729,252
Chr5	97	108,710,392
Chr6	168	139,701,585
Chr7	149	77,327,890

### Genome annotation

A combined strategy of homology alignment and *de novo* search was applied to identify repetitive elements in the genome. Tandem repeats were detected using Tandem Repeats Finder (TRF) v4.09^32^. Repetitive elements homologous to those available in the Repbase28.06^33^ were identified with RepeatMasker v4.1.0 and RepeatProteinMask v4.1.0^34^. In addition, a *de novo* repetitive elements database was generated using LTR_FINDER v1.0.6^35^, RepeatScout v1.0.5^36^, and RepeatModeler v2.0.1^37^. The resulting repeat sequences with lengths greater than 100 bp and gap ‘N’ less than 5%, obtained from both two strategies, were combined to construct the raw transposable element library. This library was then processed by UCLUST algorithm^38^ to yield a non-redundant library, followed by DNA-level repeat identification using RepeatMasker v2.0.1^37^. The results indicated that the genome contained 52.78% repetitive elements, most of which were long terminal repeat (LTR) retrotransposons, representing 40.05% of the genome (Table 4).

**Table 4:** Summary of repetitive sequences identified in the genome of *Arma custos*.

Repeat family	*De novo* + Repbase		TE Proteins		Combined TEs	
	Length (bp)	% of genome	Length (bp)	% of genome	Length (bp)	% of genome
DNA transposon	35,910,229	3.71	4,460,637	0.46	38,432,048	3.97
LINE	51,833,697	5.35	50,304,093	5.19	84,751,997	8.75
SINE	727,401	0.08	0	0	727,401	0.08
LTR	386,285,131	39.86	22,523,095	2.32	388,095,502	40.05
Unknown	45,755,684	4.72	222	0	45,755,906	4.72
Total (TRF not included)	502,619,694	51.86	77,280,191	7.97	505,079,672	52.12

For non-coding RNA (ncRNAs) annotation, the transfer RNAs (tRNAs) were predicted using tRNAscan-SE v1.4^39^. As ribosomal RNAs (rRNAs) are highly conserved, they were predicted by searching against selected rRNA sequences from closely related species as references using the BLAST v2.2.26^40^. Other ncRNAs, including micro RNAs (miRNAs) and small nuclear RNAs (snRNAs), were identified by searching against the Rfam database v14.1^41^ using the Infernal v1.1.2^42^. Overall, 20,337 tRNAs, 1,556 rRNAs, 2,790 miRNAs and 596 snRNAs were predicted, resulting in a total of 25,279 ncRNAs (Table 5).

**Table 5:** Summary of non-coding RNAs predicted in the genome of *Arma custos*.

Class	Type	Number	Average length (bp)	Total length (bp)	% of genome
miRNA		2,790	125.08	348,971	0.036009
tRNA		20,337	73.44	1,493,526	0.15
rRNA	rRNA	778	207.06	161,090	0.016622
	18 S	214	244.99	52,428	0.00541
	28 S	477	211.16	100,722	0.010393
	5.8 S	2	110	220	0.000023
	5 S	85	90.82	7,720	0.000797
snRNA	snRNA	298	131.52	39,193	0.004044
	CD-box	46	141.17	6,494	0.00067
	HACA-box	16	187.44	2,999	0.000309
	splicing	231	124.84	28,837	0.002976
	scaRNA	5	172.6	863	0.000089

A combined three-pronged strategy, involving *de novo* prediction, homology-based gene prediction, and transcriptome-based prediction, was employed to annotate the genes in the genome. *De novo* gene models were generated by multiple programs, namely Augustus v3.3.3^43^, GlimmerHMM v3.0.4^44^, SNAP v2013.11.29^45^, Geneid v1.4^46^, and Genscan v1.0^47^. For homology-based prediction, protein sets from five bugs including *Halyomorpha halys*^48^, *Nesidiocoris tenui*^49^, *Oncopeltus fasciatus*^50^, *Rhodnius prolixus*^51^ and *Apolygus lucorum*^52^ were downloaded from Insectbase 2.0^53^ on January 3, 2022. These protein sets were aligned to the assembled genome using TblastN v2.2.26^40^ with an E-value threshold of ≤1e^−5^. The matching proteins from these bugs were used to predict the gene structure of the assembled genome with GeneWise v2.4.1^54^. For transcriptome-based prediction, raw reads from five transcriptomic libraries were subjected to quality control with fastp v0.21.0^21^. After eliminating adapter sequences and low-quality reads with Trimmomatic v1.4^55^, clean data were assembled into transcripts using Trinity v2.11.0^56^ and StringTie2 v2.1.6^57^. The candidate coding regions in these transcripts were predicted using TransDecoder v5.5.0^56^, which is implemented in the Trinity software. The resulting protein sequences were used to predict the gene structures following the procedure as described for homology-based prediction. In addition, the clean transcriptomic data were aligned to the assembled genome using HISAT2 v2.2.1^58^ to identify the exons and splice sites, and these were used to extract the gene structures using PASA v2.4.1^59^. A non-redundant reference gene set was generated by merging genes predicted by the three strategies with EVidenceModeler (EVM) v1.1.1^60^. The gene models were further updated with PASA v2.4.1^59^ to identify untranslated regions. Finally, the final comprehensive gene set was generated, resulting in a total of 13,708 protein-coding genes (Table 6). These genes had an average gene length of 25,698.42 bp. The average lengths of their coding sequence (CDS), exon, and intron length were 1,400.68 bp, 192.17 bp, and 3,863.69 bp, respectively. On average, each gene contained 7.29 exons (Table 7).

**Table 6:** Summary of protein-coding genes annotated in *Arma custos* genome by three strategies.

	Gene set	Number	Average transcript length (bp)	Average CDS length (bp)	Average exons per gene	Average exon length (bp)	Average intron length (bp)
*De novo*	Augustus	19,761	14,722.95	1,082.37	5.26	205.61	3,198.78
	GlimmerHMM	58,896	10,708.91	415.56	3.21	129.28	4,648.56
	SNAP	13,204	64,327.32	600.21	9.76	61.49	7,274.35
	Geneid	17,994	24,118.89	970.43	4.28	226.78	7,059.29
	Genscan	19,234	31,631.85	1,038.01	5.3	195.91	7,117.62
Homolog	Nten	7,909	8,905.95	957.12	4.39	218.25	2,347.9
	Aluc	11,081	11,029.78	1,076.08	5.24	205.26	2,346.15
	Hhal	15,287	12,849.29	1,176.33	5.82	202.21	2,423.1
	Ofas	14,804	5,945.94	795.81	3.76	211.46	1,863.76
	Rpro	11,814	8,474.72	932.33	4.63	201.46	2,079.04
Transcriptome	PASA	20,975	25,353.77	1,278.69	6.62	193.13	4,283.15
	Transcripts	35,938	44,647.4	2,807.63	8.33	336.92	5,705.45
EVM		19,234	17,188.62	1,098.45	5.64	194.8	3,468.63
PASA update		19,029	20,359.35	1,128.47	5.79	195.06	4,018.72
Final set		13,708	25,698.42	1,400.68	7.29	192.17	3,863.69

Nten, *Nesidiocoris tenui*; Aluc, *Apolygus lucorum*; Hhal, *Halyomorpha halys*; Ofas, *Oncopeltus fasciatus*; Rpro, *Rhodnius prolixus*.

**Table 7:** Comparison of protein-coding genes annotated in the genomes of *Arma custos* and other bugs.

Species	Number	Average transcript length (bp)	Average CDS length (bp)	Average exons per gene	Average exon length (bp)	Average intron length (bp)
Acus	13,708	25,698.42	1,400.68	7.29	192.17	3,863.69
Rpro	15,438	7,353.42	1,059.56	5.77	183.5	1,318.33
Ofas	19,587	11,934.86	899.86	5.09	176.68	2,695.92
Aluc	20,111	22,559.88	1,348.05	6.6	204.18	3,786.2
Nten	24,514	7,117.77	957.91	4.22	226.79	1,910.8
Hhal	14,454	22,935.31	1,445.21	7.39	195.44	3,360.68

Achi, *Arma custos*; Rpro, *Rhodnius prolixus*; Ofas, *Oncopeltus fasciatus*; Aluc, *Apolygus lucorum*; Nten, *Nesidiocoris tenui*; Hhal, *Halyomorpha halys*.

The annotation of the protein-coding genes was performed using BLAST v2.2.26^40^ against SwissProt and National Center for Biotechnology Information (NCBI) non-redundant (Nr) database with DIAMOND v2.2.22^61^, parameters used ‘-ultra-sensitive -max-target-seqs. 1 -evalue 1e^−5^’ with a threshold of E-value ≤ 1e^−5^. The motifs and domains present in the predicted proteins encoding by these genes were annotated using InterProScan v86.0 with parameters ‘-disable-precalc, -goterms, -pathways’ and Pfam^62^. Additionally, these genes were classified into functional categories based on KEGG^63^ and GO^64^ with a threshold of E-value ≤ 1e^−5^. Overall, 13,219 predicted genes were annotated using the databases of Nr, SwissProt, InterProScan, Pfam, KEGG and GO, representing 96.43% of the total gene set (Table 8).

**Table 8:** Summary of functional annotation of protein-coding genes encoded in genome of *Arma custos*.

Database	Number	Percent (%)
Nr	12864	93.84
Swissprot	9933	72.46
InterPro	12353	90.12
Pfam	9729	70.97
KEGG	10228	74.61
GO	7810	56.97
Annotated at least one database	13219	96.43
Unannotated	489	3.57
Total	13708	

## Data Records

The raw data of Illumina short reads, PacBio HiFi long reads and Hi-C reads for assembling the genome of *A. custos*, as well as the transcriptome Illumina sequencing data for genomic annotation, have been deposited in the NCBI SRA (Sequence Read Archive) database under BioProject number PRJNA1001510. Illumina sequencing data for genome survey can be accessed and downloaded with accession number SRR25498178^65^. PacBio sequel II sequencing data for genome assembly can be accessed and downloaded with accession number SRR25503034^66^. Hi-C sequencing data can be accessed and downloaded with accession number SRR25518321^67^. Transcriptome sequencing data for genome annotation can be accessed and downloaded from NCBI SRA database (https://identifiers.org/ncbi/insdc.sra:SRP453032)^68^. The genome sequence has been deposited in Genbank under the accession number JBBAGI000000000 (https://www.ncbi.nlm.nih.gov/nuccore/JBBAGI000000000)^69^. The final chromosome assembly, genome structure annotation, amino acid sequences and CDS sequences data are available at the Figshare database (10.6084/m9.figshare.25284943)^70^.

## Technical Validation

The accuracy of the assembled genome was assessed using two methods. Firstly, the clean Illumina genomic short reads were aligned back to the genome by Burrows–Wheeler Aligner (BWA) v.0.7.12-r1039^71^. Approximately 97.81% of the short reads were successfully aligned to the genome, providing a genome coverage of 99.95%. The heterozygous and homozygous nucleotide polymorphisms (SNPs) in the genome were 0.407191% and 0.00011%, respectively. The results indicate a high accuracy of the genome assembly. Secondly, the accuracy of the assembled genome was evaluated using Merqury v1.4^72^. A quality value of 46.78 was obtained, affirming the base-level accuracy genome assembly. The completeness of the assembled genome was evaluated using three methods. Firstly, Benchmarking Universal Single-Copy Orthologs (BUSCO) v5.4.7 (-l insecta_odb10 -m genome)^73^ was employed. The results showed that the complete and fragment scores were 99.34% and 0.22%, respectively. Among the retrieved complete single-copy genes, only 2.3% of them are duplicated. Secondly, Core Eukaryotic Genes Mapping Approach (CEGMA, v2.5)^74^ was employed. Among the 248 most highly conserved core eukaryotic genes (CEGs) within CEGMA, 230 CEGs were successfully assembled, accounting for 92.74%, and 222 CEGs were complete, accounting for 89.52%. Thirdly, LTR Assembly Index (LAI) was assessed using LTR_retriever v. 2.9.0^75^, resulting in a value of 8.44. These results indicated a high level of completeness in the genome assembly.

## Data Availability

In this study, no custom scripts or command lines were utilized. All software employed for data processing and analysis are publicly available. The specific versions and parameters of each software are detailed in the Methods section. If no specific parameters were mentioned for a particular software, default parameters were used. The software was applied following the manuals and protocols provided by the respective bioinformatic tools.
